# Dual-Sensing Piezoresponsive Foam for Dynamic and Static Loading

**DOI:** 10.3390/s23073719

**Published:** 2023-04-04

**Authors:** Ryan A. Hanson, Cory N. Newton, Aaron Jake Merrell, Anton E. Bowden, Matthew K. Seeley, Ulrike H. Mitchell, Brian A. Mazzeo, David T. Fullwood

**Affiliations:** 1 Department of Mechanical Engineering, Brigham Young University, Provo, UT 84602, USA; toorah26@byu.edu (R.A.H.); cory.newton@gmail.com (C.N.N.); jake.merrell@xonano.com (A.J.M.); abowden@byu.edu (A.E.B.); 2 Department of Exercise Science, Brigham Young University, Provo, UT 84602, USA; matthewkseeley@gmail.com (M.K.S.); rike_mitchell@byu.edu (U.H.M.); 3 Department of Electrical and Computer Engineering, Brigham Young University, Provo, UT 84602, USA; bmazzeo@byu.edu

**Keywords:** nanocomposite, piezoelectric, piezoresistive, multifunctional

## Abstract

Polymeric foams, embedded with nano-scale conductive particles, have previously been shown to display quasi-piezoelectric (QPE) properties; i.e., they produce a voltage in response to rapid deformation. This behavior has been utilized to sense impact and vibration in foam components, such as in sports padding and vibration-isolating pads. However, a detailed characterization of the sensing behavior has not been undertaken. Furthermore, the potential for sensing quasi-static deformation in the same material has not been explored. This paper provides new insights into these self-sensing foams by characterizing voltage response vs frequency of deformation. The correlation between temperature and voltage response is also quantified. Furthermore, a new sensing functionality is observed, in the form of a piezoresistive response to quasi-static deformation. The piezoresistive characteristics are quantified for both in-plane and through-thickness resistance configurations. The new functionality greatly enhances the potential applications for the foam, for example, as insoles that can characterize ground reaction force and pressure during dynamic and/or quasi-static circumstances, or as seat cushioning that can sense pressure and impact.

## 1. Introduction

Nanocomposite piezoresponsive foam (NCPF) is a recently developed self-sensing material that produces a voltage during rapid deformation, and hence can be utilized to characterize impacts for applications such as helmets, sports padding, shoe insoles, and vibration-isolating components [1,2,3,4,5]. However, there is currently no consensus on what specifically is being measured by a given foam sensor; i.e., what characteristic of the deformation (energy, impulse, velocity, etc.) correlate best with the voltage that is sensed. Furthermore, given that the voltage response is elicited by dynamically straining the foam, there remains an open question as to what deformation rate is too low for the sensors to provide a meaningful response; and whether the sensors can be used to quantify quasi-static deformation or pressure, such as quantifying the weight of a user if NCPF is utilized in a sensing-shoe insole. Weight or weight distribution is difficult to isolate from existing dynamic ground reaction force data because the material is experiencing both the loading due to the impact and the user’s weight. The aim of the present study is to characterize the response of foam sensing material during cyclical deformation, across a range of different frequencies, in order to correlate voltage response with deformation characteristics, and provide insights into sensor performance at low deformation rates. Furthermore, the characteristics of polymeric nanocomposite sensors are known to be sensitive to temperature. A previous study hypothesized that this related to a change in the foam matrix modulus, which led to a higher amplitude of strain during impact for the tested load case [2]. In this study, we maintained a constant strain amplitude while the dynamic (voltage) responses of the sensors were studied across a range of temperatures, in order to determine the relevant correlations for improved interpretation of sensor responses in practical applications. Finally, the potential to sense quasi-static deformation/pressure using the same foam material was also analyzed by characterizing the resistive response of the foam sensors during deformation, in two different dimensions.

Traditional sensors used to measure dynamic and quasi-static loading include foil strain gauges, fiber-optic sensors, pressure sensors, and triaxial accelerometers [6,7,8,9]. Accelerometers are often used for measuring dynamic impact and vibration [9,10]. On the other hand, foil strain gauges [6,7] are commonly selected when measuring the quasi-static strain of engineering components, albeit, only at small strain levels. Furthermore, application to low-stiffness structures may introduce undesired measurement-induced bias [11].

Several recent studies have focused on a range of self-sensing materials, comprised of polymer composite materials, which exhibit piezoresistive responses with a much larger range of tolerable strain [12,13]. A piezoresistive response is characterized by the varying electrical resistance of a material due to strain. Deforming a polymer matrix embedded with certain nanoparticles, such as nickel-coated carbon fibers and nickel nanostrands, elicits a piezoresistive response correlated with the strain [14,15,16]. Combining multi-wall carbon nanotubes (MWCNT) with an epoxy matrix produces a direction-sensitive piezoresistive response in bending [17] and a simple response during tensile loading [18].

Composite foams have been developed with piezoresistive properties that enable sensing of compressive strain [19] and pressure [20]. A variety of matrix materials and nanoparticle fillers have been combined to produce piezoresistive materials, including polypropylene and polyurethane, nanofibers, graphene, and MWCNT [21]. As strain is applied to this class of materials, the distance between nanojunctions decreases, enabling increased quantum tunneling, a proposed cause of the extreme piezoresistive effect [22,23].

Polymer foams are ideal for self-sensing in many applications because they can provide tailored levels of compliance and damping in mechanical systems. NCPF materials have been developed that undergo a constriction or expansion when a high voltage is applied [24]. The reverse effect—a charge arising from deformation—has only recently been discovered with nanocomposite foams that exhibit the quasi-piezoelectric (QPE) phenomenon during impact [1,2]. These NCPF materials have been tailored for use in a variety of applications including vibration monitoring [1], measuring ground reaction force in shoe insoles during running and walking [3,4], and detecting impact magnitude in football helmets [5].

The present work describes a dual-response nanocomposite piezoresponsive foam (NCPF) material that exhibits both a quasi-piezoelectric (QPE) response in response to dynamic deformation, as well as a simultaneous quasi-static piezoresistive (QPR) response. The dual-response nature of the NCPF presents opportunities for self-sensing applications where low-frequency loading may be interrupted by periods of high-frequency loading (e.g., weight-distribution tracking insoles or pressure-sensing seat cushions), as well as distinguishing between high-frequency loads that have distinct imposed constant loads (e.g., insoles that can compute both dynamic and static load contributions to energy expenditure).

## 2. Materials and Methods

**Figure 1:**
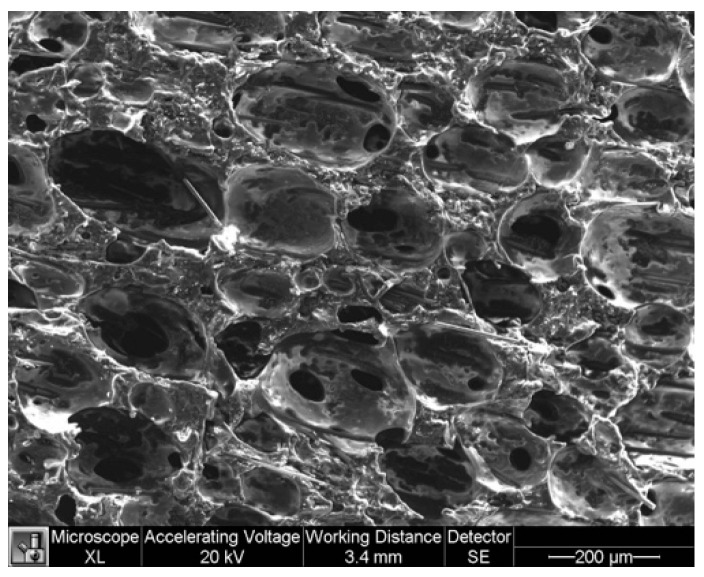
SEM image of closed cell NCPF structure, containing conductive particle additives.

Nanocomposite foams made from several different polymer matrix materials have been shown to exhibit quasi-piezoelectric effects [25]. A polyurethane-based foam (AFX-20655 Polyurethane from Utah Foam Products) was selected for this study because it has characteristics similar to typical shoe insoles that are applications of interest; it is also readily available and economical. Polyurethane-based sensors are made by casting the low-density foam into a custom aluminum mold. To prepare the foam mixture, conductive particles were incorporated into the polyol or ‘B-side’ component of the two-part foam base until the additives were well dispersed. The conductive particles were comprised of 15% nickel nanoparticles (Novamet 526LD) and 5% 1 mm long nickel-coated carbon fibers (from Conductive Composites) by weight of total mixture, based on previous optimization studies. Previous iterations of the foam mixture, which only provided the QPE response but lacked a piezoresistive characteristic, used conductive particles comprised of 5–7% nickel nanoparticles and 1–2% nickel-coated carbon fibers by weight of total mixture. The isocyanate or ‘A-side’ component was then added to this mixture, rotated in a planetary centrifugal mixer, and then poured into the mold, which was already heated to 45 °C. Once in the mold, a metal plate was clamped over the opening. The foam was left to cure for 15 min on a hot plate, still at 45° C, after which the partially cured foam was removed from the mold and left to sit at room temperature for 24 h to finish the curing process. The specimens had a final density in the range of 200–350 kg/m^3^. The foam is a closed cell foam with microstructure visible via SEM images in Figure 1. A typical cell in the NCPF foam measures between 150–200 microns in length.

### 2.1. Piezoelectric Response Measurement

For the cyclical testing, cuboid samples were manufactured with dimensions of 6.35 cm × 6.35 cm × 1.25 cm. Voltage measurements were taken by probing the samples with a four-point lance measurement probe inserted into one side (a row of 6 mm long lances, separated by 2 mm, connected in parallel), and the voltage from the probe recorded relative to the building ground.

An Instron 1321 testing system was used for lower frequency (<10 Hz) cyclical compression of the foam sensors. This is approximately the frequency limit for this range of displacement on the Instron used. Steel plates were placed above and below the sample, and were insulated with vinyl (electrical) tape to prevent electrical interference from the equipment. Samples were cyclically compressed in a sinusoidal manner to a maximum compressive deformation of 5 mm; i.e., a peak compressive strain of 0.4. A maximum compressive deformation of 5 mm was selected to avoid compression of the probe and probe housing, ensuring compression was limited to the foam itself.

**Figure 2:**
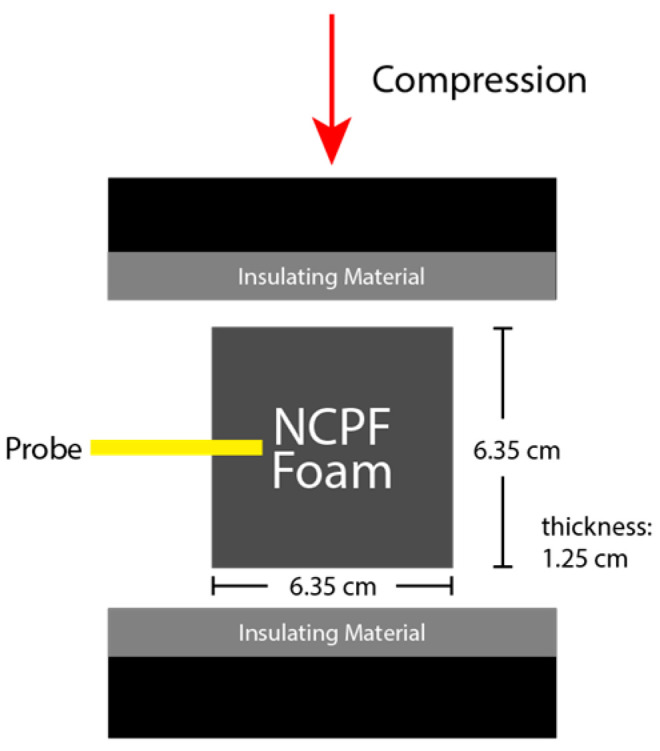
Schematic depicting experimental setup for cyclic testing NCPF samples utilizing probes to measure dynamic loading voltage response.

A cyclic compression testing apparatus was custom-built to subject the foam sensors to higher frequency cyclic compressions. The apparatus is capable of sinusoidally compressing foam sensors at frequencies between 10–45 Hz with a peak-to-peak amplitude of 5 mm (the same amplitude used in the Instron cyclic trials). A frequency of 45 Hz is the upper frequency limit of the apparatus used for the desired range of displacement. This frequency range represents a variety of industry vibrations where sensing foam could be applied. The apparatus uses two electric motors to drive a crank disc, and a connecting rod transfers the high frequency cyclic rotation into reciprocal linear displacement of a steel plate. Another steel plate was fixed above the reciprocating steel plate, and the foam sensors were compressed between the two surfaces. The plates were covered again with vinyl (electrical) tape in order to electrically isolate the foam sensor from the apparatus. Zero prestrain was introduced by setting the platform plate to bottom dead center, placing the foam sample on the platform and fixing the upper rigid plate to make non-compressive contact, followed by cycling. The experimental setup is shown in Figure 2. The reciprocating frequency of the platform plate was measured using a laser tachometer and validated via a spectrogram of the foam sensor’s voltage response.

A National Instruments 9234 Data Acquisition (DAQ) module, in conjunction with a custom LabVIEW dashboard, was used to register electrical signals emitted by the sensor as it was compressed.

### 2.2. Piezoresistive Response Measurement

In order to study the piezoresistive responses of the foam, two different sensor configurations were used, with resistances measured either through the thickness of the foam (parallel to the direction of compression) or in-plane (perpendicular to the direction of compression). For certain applications it is advantageous or convenient to use one of the two configurations. For example, the in-plane configuration is preferred for insoles where it may be difficult to provide robust electrical connection to both sides of the foam. For through-thickness samples, conductive adhesive was used to adhere strips of aluminum-coated polyimide film to opposite sides of each sample (to act as electrical contacts).

**Figure 3:**
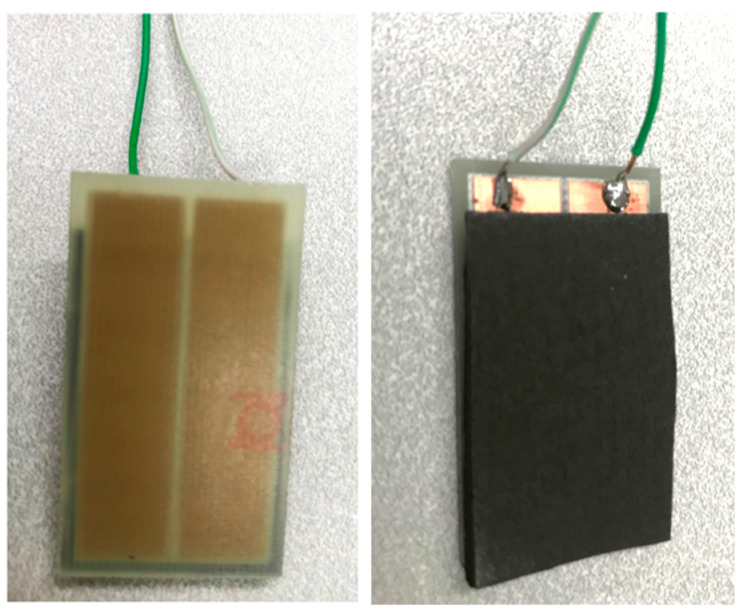
Bottom view (**left**) and top view (**right**) of sample for in-plane resistance measurement of dimension 25 mm × 40 mm.

**Figure 4:**
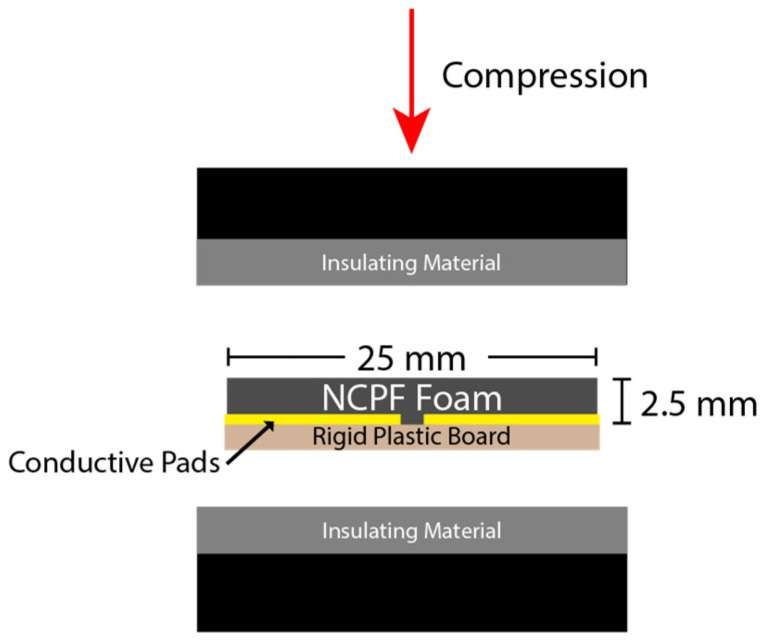
Schematic depicting experimental setup for in-plane sensor configuration to measure piezoresistive response to static loading. Direction of compression to induce static strain shown.

For in-plane samples, a 25 mm × 40 mm single-sided copper-clad laminate board was used, and a 1 mm gap was machined in the copper layer sheet to leave two separate electrical contacts. Nickel-filled conductive adhesive was then used to adhere the remaining copper pads to one side of the foam. In this way, when the samples were compressed, the gap size between the two electrical contacts remained constant (the rigid plastic board served to prevent transverse deformation between the electrical contacts due to the Poisson’s ratio). Figure 3 shows one of the in-plane samples; the left image is a bottom view showing the rigid plastic board through which the fixed copper sheets can be seen, and the right image is a top view showing the polyurethane-based foam and electrical contacts. Figure 4 shows how the in-plane sample was positioned in the Instron for compression.

**Figure 5:**
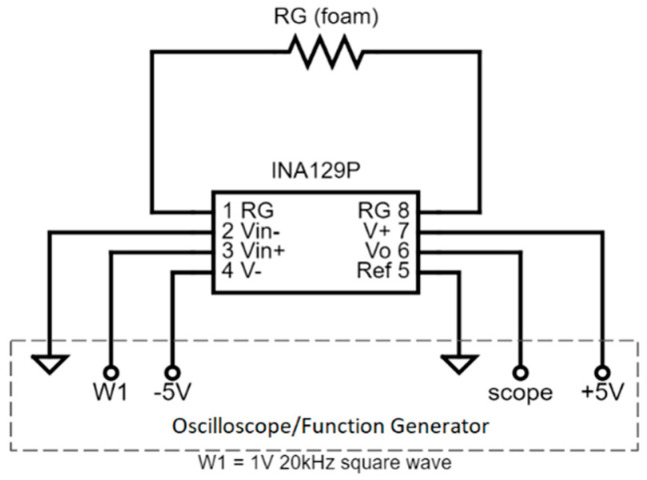
Schematic of sensor connection to oscilloscope/function generator for resistance measurements.

The samples were compressed in the Instron to a maximum 0.7 strain to avoid foam densification, which occurs around 0.75–0.8 strain [26]. An oscilloscope and function generator provided an alternating input voltage (±3 V). The oscilloscope was connected to the sensor using an instrumentation amplifier (INA129P). The foam sensor served as the gain resistor in the instrumentation amplifier circuit as shown in Figure 5.

The amplifier gain (*G*) was measured, before the resistance of the sensor (*R_G_*) was calculated from the gain equation:

(1) $$G=1+\frac{49.4k\Omega }{R_{G}}$$

The voltage was measured at a sampling rate of 10 Hz with no smoothing. Measurements were taken several seconds after applying a given compression load; i.e., static loading conditions. The accuracy of the measurement setup was verified by measuring the resistance of several known resistors.

## 3. Results

### 3.1. Piezoelectric Response to Cyclic Compression

**Figure 6:**
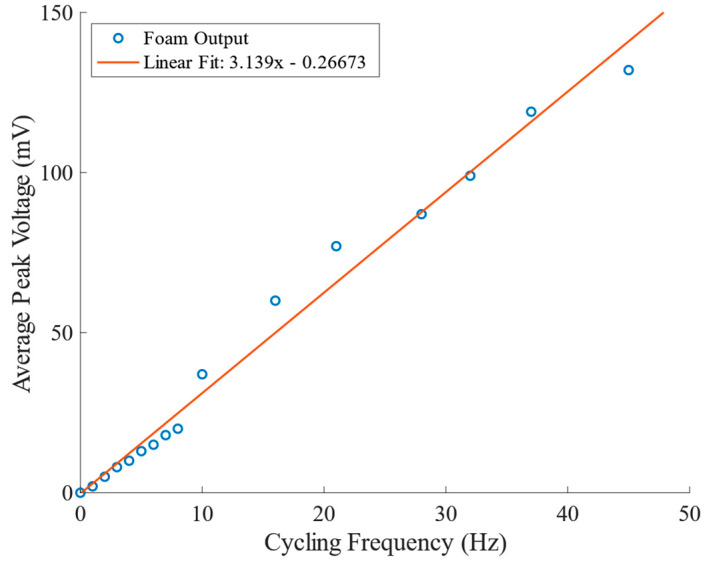
Average peak voltage response versus cycling frequency, 1–45 Hz.

Figure 6 combines voltage vs frequency data from all cyclical compression tests, plotting the average peak voltage at each frequency; the Instron tensile testing machine was used for 1–8 Hz data points, and the cyclic compression testing apparatus was used for frequencies 10–45 Hz. The quasi-piezoelectric voltage response of the nanocomposite foam sensors increases linearly with compression frequency, with R^2^ = 0.986.

In order to reduce the drift from the temperature–voltage/foam modulus effect for the higher frequency tests, the sensor was held for 300 s at 45 Hz to reach a steady-state temperature and voltage responses; the frequency was then decreased in steps, and held at 37 Hz, 32 Hz, 28 Hz, 21 Hz, 16 Hz, and 10 Hz for 15 s each time. Lower frequency testing was then performed on the Instron tensile test machine. As the change in temperature was negligible for the frequencies achieved on the Instron, the low-speed tests were performed in ascending order, with cycling applied in 1 Hz increments from 1 to 8 Hz, and with a 10 s dwell at each frequency; however, the samples were therefore at different temperatures for the two sets of tests, and this likely caused the small change in the slope between the 0–10 Hz data and the higher frequency data in Figure 6.

The figure also indicates that the voltage response of the sensors is positively correlated to maximum velocity or impulse—both of which also vary linearly with frequency during cyclical testing. The dependence of voltage amplitude on peak velocity would imply that for a fixed amplitude, the voltage increases linearly with frequency, as observed; conversely, for a fixed frequency, strain amplitude would be the physical quantity relating directly to the measured voltage.

**Figure 7:**
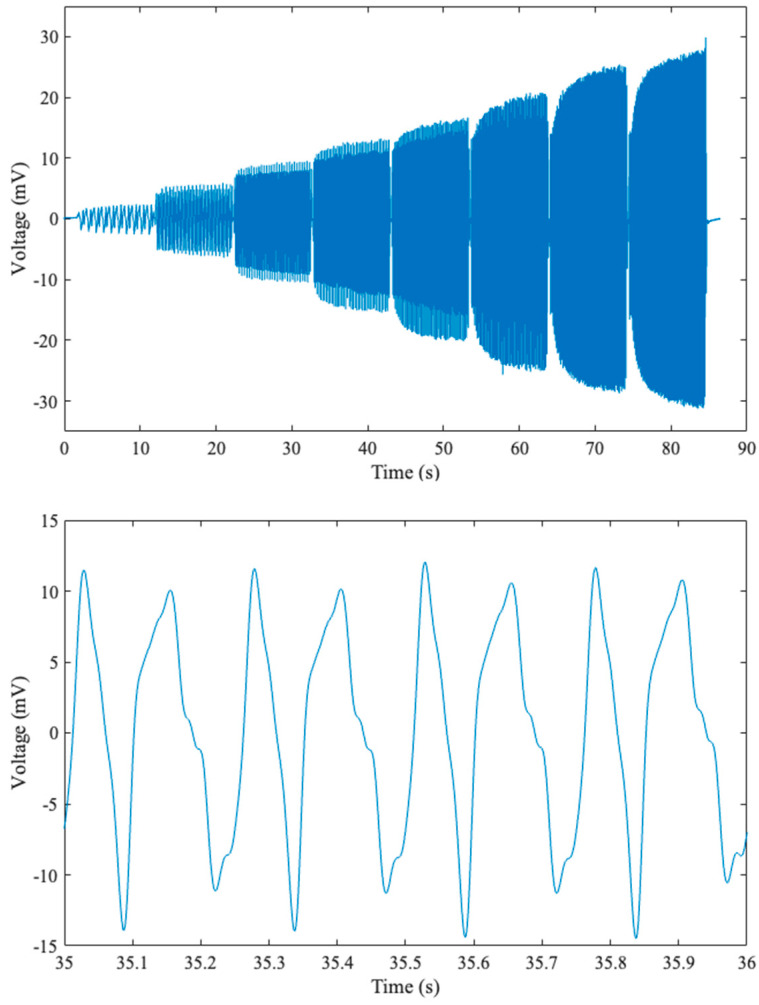
Voltage response versus time for 1 to 8 Hz cyclical testing in the Instron (**top**) and a zoomed-in version of the 4 Hz voltage response (**bottom**).

Figure 7 (top) shows the actual voltage response across the 0–8 Hz compression range over a period of about 90 s. The bottom plot in the figure shows a zoomed-in plot of cyclic compressions at 4 Hz.

**Figure 8:**
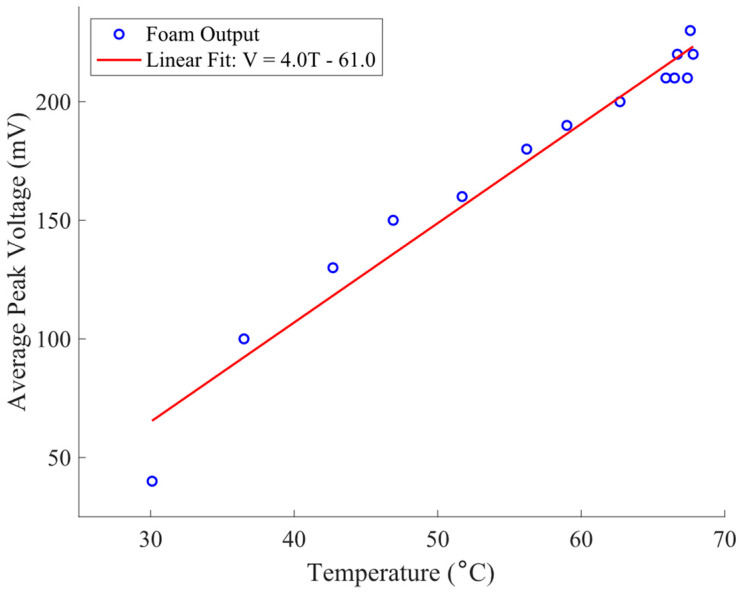
Average peak voltage versus temperature over 130 s for 45 Hz cyclical compression.

To characterize the effect of temperature on voltage, the cyclic compression apparatus was used to apply compression to a sensor sample of smaller dimension at a single frequency (45 Hz) for 130 s, and the temperature and voltage were recorded at intervals. Both temperature and peak voltage increased over time. The results are shown in Figure 8, which shows a linear correlation between peak voltage and temperature.

### 3.2. Piezoresistivity Characterization

**Figure 9:**
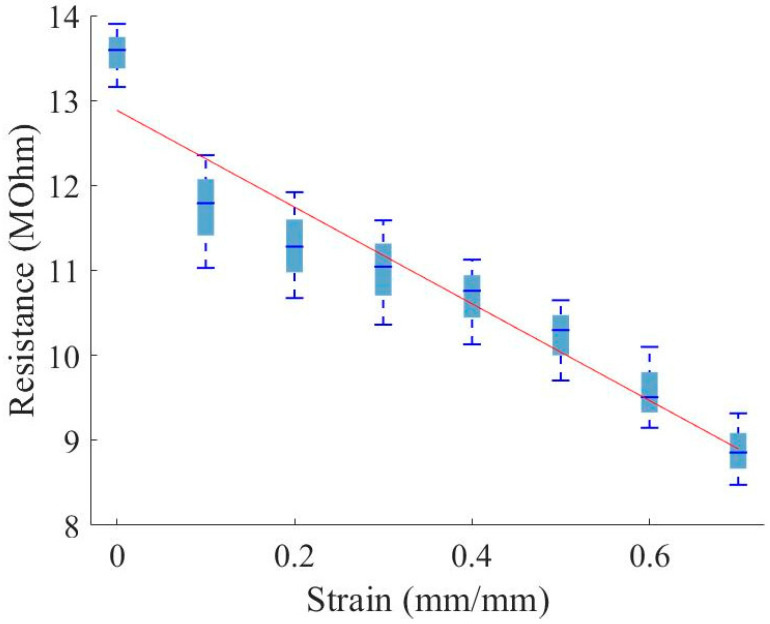
Distribution of resistance versus strain; resistance measured through the material thickness.

**Figure 10:**
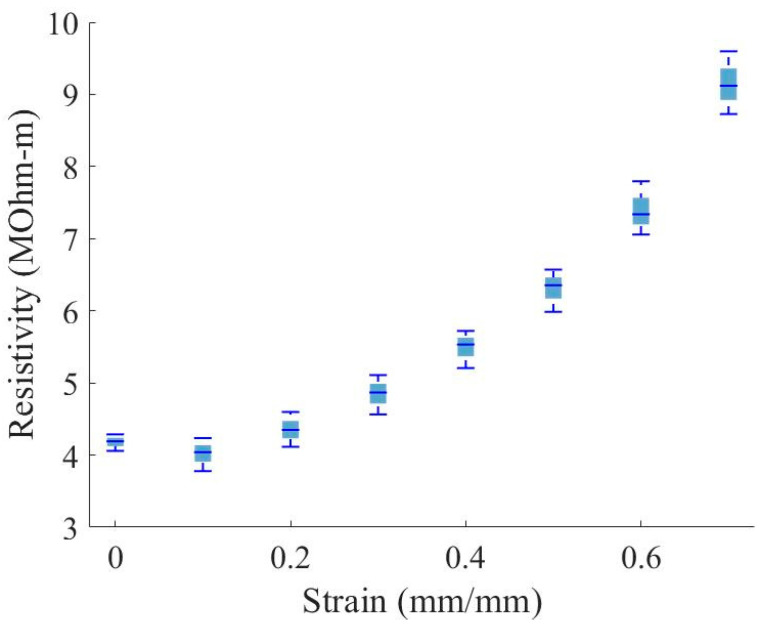
Distribution of material resistivity versus strain measurements; resistance measured through the material thickness.

To characterize the piezoresistive response of the nanocomposite foam, tests were initially performed on three through-thickness samples to measure the resistance through the material under compressive strain. Each sample was compressed to several strain levels while measuring the resistance across the foam. Figure 9 plots the resistance versus strain for the through-thickness samples, and Figure 10 plots the calculated bulk material resistivity versus strain of four through-thickness samples. Bulk resistivity (ρ) is calculated at each strain level using the measured resistance values (R), the contact surface area of the sample at the electrical contacts (A), and the distance between the electrical contacts (L). Assuming negligible contact resistance between the copper plates and foam sample, the equation used is:

(2) $$\rho =\frac{RA}{L}\;\mathrm{or}\;R=\frac{\rho L}{A}$$

While the resistance measured through the sample thickness decreases with increased strain (Figure 9), the material bulk resistivity increases with increased strain (Figure 10). This finding indicates that the decrease in resistance with increased strain is primarily due to the change in distance between the electrical contacts. Note that the initial change in resistance between zero and 0.1 strain may relate to a change in contact resistance of around 1.5 MOhm.

**Figure 11:**
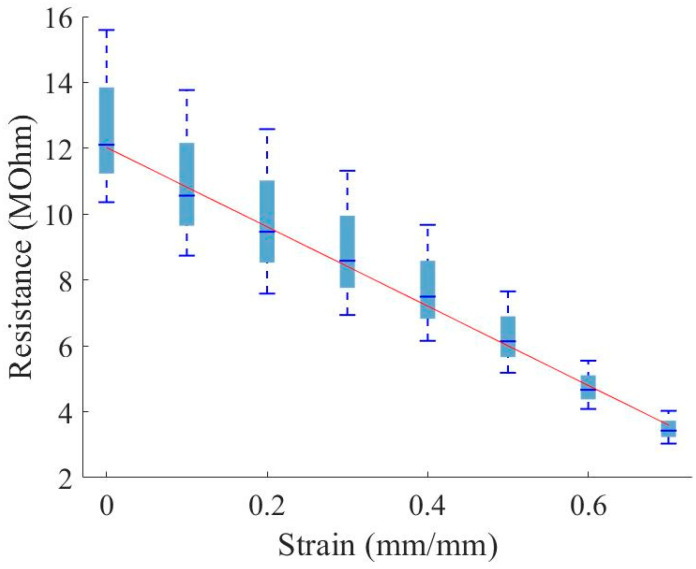
Resistance versus strain; resistance was measured across a 1 mm in-plane gap between copper strips.

Figure 11 plots resistance versus strain for the in-plane samples. For each sample the resistance decreases approximately linearly with increased strain. However, given that the resistivity increased in the through-thickness samples, one would naturally expect that the resistance would increase for the in-plane samples since the distance between the conductors did not change. This indicates electrical anisotropy in the foam during the compression—either present in the as-manufactured material or developing during compression. During compression, cell walls in closed-cell polymeric foams are known to stretch perpendicular to the compressive load, while the edges of the cell wall bend [27]. This cell deformation pattern would cause the nickel-coated carbon fibers in the composite foam to realign transversely to the compression axis. It is hypothesized that this realignment of fibers causes the observed anisotropic resistance response. Note that this discussion is based upon the assumption of a low contact resistance, as mentioned above.

**Figure 12:**
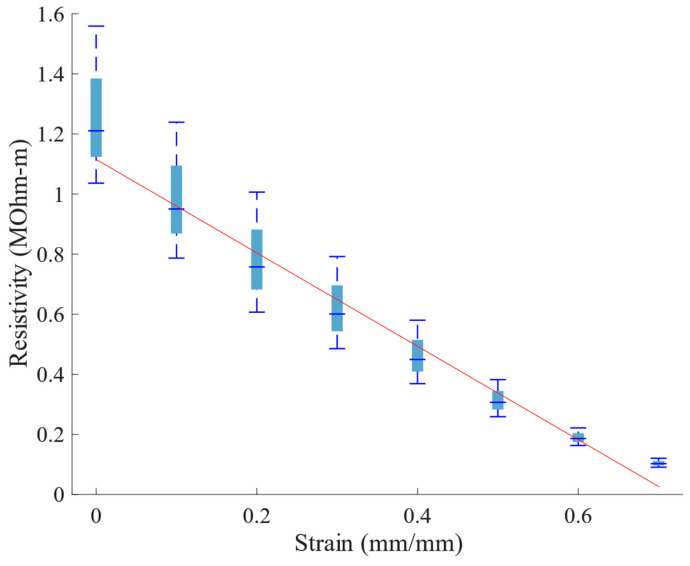
Resistivity versus strain; resistance was measured across a 1 mm in-plane gap between copper strips.

To aid the discussion of through-thickness vs in-plane response, Figure 12 plots resistivity versus strain for the in-plane samples based upon a simplifying assumption that the current that travels between the two resistors is evenly distributed across the cross-section of the foam. Clearly, this is not likely to be the case since the current will be higher closer to the surface with the contact; however, the calculation based upon the simplifying assumption can nevertheless be enlightening and place a boundary on the actual resistivity of the material. From the figure, it is clear that the resistivity calculated in this manner drops rapidly with strain, in a linear fashion, while the resistivity in the through-thickness direction increases with strain. This supports the discussion regarding the significantly different resistivity response in the through-thickness vs. the in-plane directions under strain, as discussed above.

## 4. Discussion

The results indicate that the maximum voltage associated with cyclical deformation of the foam (at constant temperature) is linearly related to the rate of deformation (or maximum velocity of the foam surface during compression; Figure 6). Previous studies on similar foams have hypothesized that the piezoelectric response is caused by a triboelectric electric effect occurring between particles and within cells of the foam [5]. Based upon the underlying equations for triboelectricity, a proportional relationship between deformation rate and voltage would be expected (see the development of such equations in [28]). A similar example of such a relationship is demonstrated in an experimental study of nanogenerators [29]. In the case of that particular study, voltage increased with rate up to the point where the nanogenerator could not recover fully from the deformation of one cycle before the next cycle began. In the case of the foam of the current study, it appears that it is able to fully recover between cycles all the way up to 45 Hz, for the applied strain of 0.4.

It is interesting that the voltage also increases with temperature (Figure 8). Previous studies on the effect of temperature on the triboelectric effect between surfaces indicate that the effect diminishes at a higher temperature [30]. The authors report that in previous studies, a ~20% decrease in voltage generated was observed for the reported system (Al and PTFE) across a similar range of temperatures. They also develop a model to explain such a drop in voltage. Hence, it appears that temperature dependence of the actual triboelectric effect itself does not explain the increase in voltage with temperature shown in Figure 8. An alternative explanation is that the increase in temperature reduces the modulus of elasticity of the polyurethane material, leading to more rapid collapse of the cells within the foam, and thereby increasing the voltage for the same reason that higher frequencies increase voltage. While we cannot report accurate values of temperature dependence for the foam being tested in this study, various studies of rigid polyurethane foams have demonstrated reductions in the modulus of 25% or more for temperature increases from 20 °C to 70 °C (the temperature increase observed for compression at a steady frequency of 45 Hz in Figure 8) [31,32].

On the other hand, if lowering the modulus increases the rate of internal collapse, and associated triboelectric effect, then increasing the frequency of cyclical deformation might also be expected to increase apparent stiffness, thereby lowering (at least to some extent) the voltage produced. For two different classes of polyurethane foams, the increase in the modulus when increasing cyclical deformation from 0 Hz to 45 Hz has been shown to be of the order of ~2 [33,34]. However, even combining this potential increase in the modulus with the potential drop in voltage associated with a modulus drop, as indicated by Figure 8, the effect is orders of magnitude less than that associated with the change in frequency over the studied frequency range, as indicated in Figure 6.

In terms of the resistivity of the foam, tests that were performed to determine the sensitivity of the resistance response to temperature did not show a statistically meaningful change in resistance with temperature between room temperature and 60 °C, and hence were not included in this paper. This is consistent with the resistance phenomenon being hypothesized to be dominated by quantum tunneling [16,23]; we have not found a strong connection between temperature and quantum tunneling in the literature for relatively small temperature changes such as those studied here.

The lowering of the resistance in the through-thickness direction (Figure 9) is presumably dominated by the decrease in distance between the conductors, while the resistance decrease along the in-plane direction (Figure 11) can be explained by the densification of conductive material in the region near the conductors, during compression. This densification naturally leads to the anisotropic nature of the resistivity, as displayed in Figure 10 and Figure 12; as cells within the foam are collapsed by the compressive load, the linear density of the conductive cell walls increases normally compared to the conductor surface increases, while the linear density in a direction parallel to the surface does not change significantly.

In this study, the QPE and QPR responses were measured independently of one another. In a clinical situation (such as monitoring weight shifting of a knee replacement patient during one exercise, and monitoring gait during a different exercise), it may be reasonable to expect that the two modes are monitored independently—during different time periods. However, in a real life application, any dynamic deformation of the sensor (i.e., QPE response) may affect the accuracy of the QPR response if monitored simultaneously. Hence, it is assumed that the two different electrical circuits will be probed over different time periods (presumably in an alternating manner). The QPE response (voltage measured) approaches zero as the strain rate approaches zero. If any impact or significant strain rate is observed via the QPE response, the QPR response would not be probed until the QPE response is no longer significant.

## 5. Conclusions

The purpose of this paper was to characterize the dual-response quasi-piezoelectric/quasi-piezoresistive responses of a self-sensing NCPF. The dual-response characteristics facilitate quasi-static pressure and dynamic force sensing from the same NCPF sensors. In particular, we demonstrated that a polyurethane-based polymer, embedded with both nickel-coated carbon fiber and Novamet nickel powder nanoparticle additives, could produce such a configuration.

The quasi-piezoelectric voltage response of nanocomposite foam sensors increases linearly with compression frequency, for a constant amplitude. This relationship was observed for frequencies from 1–45 Hz, and indicates a linear relationship between maximum velocity and the voltage produced. The voltage response of nanocomposite foams was shown to also increase linearly with increasing temperature during cycling.

The polyurethane base polymer, when embedded with an increased conductive-nanoparticle content, was found to be capable of displaying piezoresistivity, in that the material resistivity changes with increased strain. Interestingly, the resistivity change with respect to strain was anisotropic: resistivity increased with respect to strain when measured through the thickness of the material and decreased with respect to strain when measured in-plane (in the direction perpendicular to strain). We hypothesize that this may be influenced by a direction-biased nickel-coated carbon fiber realignment within the structure of the NCPF material during compression, particularly due to the known phenomenon of anisotropic closed-cell foam deformation, although verification of that hypothesis is beyond the scope of the present work. In both cases, the relationship between resistance and strain was found to be approximately linear. The existence of piezoresistivity in the QPE foam enables the measurement of quasi-static deformation, or pressure on the foam, providing a new, additional functionality.

With this new dual-responsive capability, the foam can be used to measure both dynamic and quasi-static deformation. This greatly expands the functionality of this self-sensing foam in many applications. The foam sensing material might be tailored to detect long-term compression and short-term impacts experienced by components such as gaskets, human contact points with vibration-prone equipment such as snowmobiles and off-road vehicles, running shoes, and spinal disc implants. In the case of a gasket on an automobile, for example, the QPE response could provide feedback on vibrations and associated fatigue life, while the QPR response could provide feedback on the pressure and associated seal. Both responses are important in assessing a complete evaluation of gasket performance. NCPF foam’s dual-sensing capabilities may reduce the complexity and number of required sensors in many future applications.

It may be instructive to compare QPR performance of the NCPF foam to traditional pressure gauges. A typical commercially available pressure gauge would provide a more linear response to pressure [35], with much less variability, than the foam sensing material. However, the advantage of the foam is the self-sensing capability (the sensor provides both a structural and a sensing capability, with the stiffness of the foam being tunable to a given requirement), along with the low cost of the foam sensing system.

Future studies have commenced to evaluate the performance of the NCPF foam in various applications, such as a load-sensing shoe insole. Foam is the native material of most shoe insoles making it a prime candidate to be replaced by a smart NCPF foam sensor array. The aim of the studies is to show that utilizing data provided by these sensors will improve quality of care in healthcare and physical therapy settings. NCPF foam insoles provide data on loading of the lower extremities anytime that a patient is wearing their shoes, increasing the quantity and precision of patient biomechanics data available to professionals overseeing care.

## Data Availability

Data created and discussed is available publicly via GitHub repository. https://github.com/toorah26/Dual-sensing-Piezoresponsive-Foam-for-Dynamic-and-Static-Loading.git (accessed on 12 February 2023).
